# Mechanical Characterization of Dicyclopentadiene and Glass-Fibre-Reinforced Polymer Subjected to Low to High Strain Rate

**DOI:** 10.3390/polym17060715

**Published:** 2025-03-07

**Authors:** Rogério F. F. Lopes, Daniela Azevedo, Gonçalo P. Cipriano, Tiago M. R. M. Domingues, Pedro M. G. P. Moreira

**Affiliations:** 1 INEGI, Institute of Science and Innovation in Mechanical Engineering and Industrial Engineering, Campus da FEUP, R. Dr. Roberto Frias 400, 4200-465 Porto, Portugal; rflopes@inegi.up.pt (R.F.F.L.); dazevedo@inegi.up.pt (D.A.); tdomingues@inegi.up.pt (T.M.R.M.D.); 2 Department of Mechanical Engineering, Faculdade de Engenharia, Universidade do Porto, R. Dr. Roberto Frias s/n, 4200-465 Porto, Portugal

**Keywords:** DCDP, GFRP, high strain rate, crashworthiness, SHPB, mechanical characterization

## Abstract

This work provides a detailed description of the procedures employed to characterize the mechanical behaviour of two materials present in a coach’s exterior panels, including glass-fibre-reinforced polymer (GFRP) and neat DCPD (dicyclopentadiene)-based polymers. Tensile tests were conducted at quasi-static, intermediate [1 s^−1^, 10 s^−1^], and high strain rates [150 s^−1^, 250 s^−1^] to obtain a comprehensive understanding of their behaviour. The results indicate positive and significant dependence on the strain rate. Additionally, GFRP demonstrates superior energy absorption capacity for higher strain rates, unlike DCPD, which exhibits a higher energy absorption capacity for QS tests. In the case of DCPD, raising the strain rate to 10 s^−1^ the maximum stress was not affected but decreased the elongation at fracture. At higher strain rates, there was an increase in maximum stress alongside greater elongation. DCPD maintained consistent stiffness across all rates ranging between 2087 MPa and 2389 MPa, and the tests disclosed a failure mode characterized by numerous surface-transverse fissures. Regarding GFRP, a more pronounced variation in stiffness is observed, decreasing from 11,005 MPa to 4532 MPa at 133 s^−1^, recovering to 7288 MPa at 252 s^−1^. In addition, the maximum stress and failure elongation tends to increase with the strain rate increase. The detailed analysis of these results provides valuable insights into the mechanical behaviour of these materials under different loading conditions.

## 1. Introduction

This research arises in the scope of the presented research in the Refs. [1,2], and it is intended to study and characterize the materials and components that will be used in the development of new passive safety solutions, with a particular emphasis on the use of polymeric materials with higher levels of eco-friendliness, such as DCPD (dicyclopentadiene) polymer [3]. Refs. [1,2] present studies evaluating a coach’s ability to withstand a frontal impact, according the regulation ECE R29 [4]. The vehicle’s fairings are primarily composed of a glass-fibre-reinforced composite and DCPD polymer. This work aims to analyse both materials to assess the feasibility of replacing glass-fibre-reinforced composite components in automotive applications, particularly in crash scenarios, given DCPD’s more environmentally friendly properties. Furthermore, the findings will contribute to the material characterization needed for future numerical simulations. It is therefore meant to ensure the use of procedures and materials that have the least environmental impact and to help with the transition to a circular economy, without compromising flexibility and the capacity to adapt to market requirements [5]. This information will later serve as input for the continuation of the presented research in Ref. [6].

New urban mobility paradigms have produced new technological problems as a result of the continual quest for innovative solutions that improve weight reduction and people’s passive and active safety [5]. When considering the use of novel materials, the preference lies in selecting those with structural properties and better energy absorption [7,8]. Among the most popular options, multi-material solutions with higher strength should be employed, such as the replacement of metallic components with non-metallic materials with a higher specific strength [9,10,11]. Additionally, conventional methods for exterior panel manufacturing commonly involve stamped steel or manually laminated fibre-glass-reinforced composites [12]. These processes have various disadvantages regarding significant investment in tooling, challenges ensuring high surface quality, limitations in production speed, and environmental impacts [13].

Composite materials are made up of two primary components: the matrix and the reinforcement. The matrix is the element that holds the reinforcing components together, which are often fibres with superior mechanical qualities [14]. The composite matrix distributes stresses from external loads, absorbs energy, and shields fibres. It comprises thermoplastic and thermosetting polymers, with thermoplastic exhibiting superior toughness and a greater capacity to absorb deformation [15], and also offers better mechanical performance due to enhanced hardness and strength [16]. Thermosetting polymers are favoured in applications for their thermal stability, dimensional stability, and electrical insulation [17].

Glass fibres are the most used reinforcement in composite materials, in the automotive industry [18]. The types of glass fibres fall into two categories: lower-cost fibres suitable for general applications and premium fibres. Over 90% consist of the general-use E-glass fibres commonly employed due to their widespread versatility. E-glass fibre has various advantages, such as high tensile and compression strength and cost-effectiveness. However, it also has limitations, including a reduced modulus of elasticity or high density [19]. Table 1 summarizes some mechanical properties and characteristics of the most used glass fibres.

Table 2 presents some of the impact resistance values of composites employing distinct types of resins and reinforcement. The values expressed in Table 2 were acquired using the Charpy impact test technique from references [20,21,22,23,24], and the values from references [18,25] were obtained according the Izod impact test method. For the value obtained from reference [26], a drop height test was employed.

In the construction of coaches, sandwich panels are commonly employed. These panels consist of two thin outer layers, usually made from composite materials like fibres. However, these panels have notable limitations concerning design flexibility and structural strength [27]. In this particular situation, a chance emerges to create novel solutions for vehicle exterior panels. This involves pioneering combinations of various energy absorption technologies, blending safety, reduced weight, cost-effectiveness, and environmental consciousness in alignment with innovative concepts of urban mobility.

Tensile tests on materials are intended to be conducted from <0.1 s^−1^ up to 250 s^−1^. According to the work in [28], for tensile tests <0.1 s^−1^, conventional loading frames are used for tests in the range [0.1, 500] s^−1^, and high-speed servo-hydraulic test machines can be chosen, while for tests in the range [200, 5000] s^−1^, the split Hopkinson tension bar (SHTB) testing apparatus is used.

FRP materials typically have multiple factors affecting strain rate sensitivity, including factors such as fibre type, deposition mode, or volume fraction and even the matrix characteristics. Due to this wide variety of parameters, it is quite difficult to find information in the literature, leading to the need for in vitro characterization of the material [28]. In this field, it is possible to highlight Körber’s work [29], which states that the author Yuanming investigated the mechanical behaviour of E-glass fibre bundles under tension using SHTB and detected a significant discrepancy in modulus strength and failure strain when the material was subjected to different strain rates.

According to Chen et al. [30], who conducted tensile tests on glass fibre/epoxy laminate (G10/FR-4) with a thickness of 1 mm (arranged in five layers with 0-degree and 90-degree orientations), quasi-static strain rates are reported in the range of 2.08 × 10^−5^ s^−1^ up to 1.04 × 10^−1^ s^−1^ and high-speed dynamic tests in the range of 2.75 s^−1^ up to 115 s^−1^. The authors concluded that the glass fibre/epoxy laminate is sensitive to strain rate. Considering the quasi-static test, the tensile strength is approximately 258.94 MPa, a value that increases with the increase in the strain rate, reaching about 428.4 MPa at 104.6 s^−1^, which represents an increase of approximately 65%.

Zhang et al. [31] investigated the effect of strain rate on unidirectional GRFP laminate (E-type glass fibre and epoxy resin) in a range from 5 × 10^−5^ s^−1^ up to 317 s^−1^. They concluded that for strain rates higher than 45 s^−1^, the Young’s modulus tends to increase considerably compared to QS tests, with an observed increase of 57.7% when the strain rate reaches 245.3 s^−1^. Regarding tensile strength within the range of 20 s^−1^, the effect of strain rate is insignificant. However, for values above 20 s^−1^, the strain rate effect induces significant growth in tensile strength, with an increase of about 141% when the rate increases to 317 s^−1^. Similarly, Naik et al. [32] conducted work in the domain of plain weave laminates of E-glass/Epoxy LY556 composite, performing tensile tests on a tensile SHPB apparatus within the range of 140 s^−1^ up to 400 s^−1^. From these tests, the authors concluded that there was an increase of 65–89% in the value of tensile strength compared to QS values.

Shokrieh and Omidi [33] conducted a study on unidirectional glass-fibre-reinforced epoxy laminates, each 1 mm thick and consisting of five layers, for QS tests and intermediate strain rates ranging from 0.001 s^−1^ up to 100 s^−1^. The properties showed a tendency to increase with the increase in the strain rate. Specifically, properties such as tensile strength, Young’s modulus, and strain at failure demonstrated this trend. In summary, the authors assert that for the tested rates, dynamic strength is 1.5 times higher when compared to QS strength.

In Barré et al.’s study [34], results more closely related to the material under analysis in this research can be found. The authors investigated the dynamic behaviour of laminates (eight plies) of polyester reinforced with E-glass fibre for strain rates ranging from 10^−1^ s^−1^ up to 100 s^−1^ and stated that the dynamic elastic modulus and tensile strength tend to increase as the strain rate increases. Similarly, Ou and Zhu [35] conducted tensile tests for strain rates from QS up to 160 s^−1^ for 0.6 mm-thick glass-fibre/epoxy composite laminates (single ply), including tests with temperature dependency. Following the experimental tests, it was observed that mechanical properties such as Young’s modulus, tensile strength, and ultimate strain show clear increases when compared under QS loading conditions. However, with the incorporation of temperature, the values of Young’s modulus, tensile strength, and toughness decrease. Ahmed et al. [36] conducted a review of the tensile behaviour of fibre-reinforced composites under varying strain rates. For glass-fibre/polyester laminates, they found that the ultimate tensile strength is sensitive to strain rates between 10^−3^ s^−1^ and 1000 s^−1^, evidenced by increases of 55%.

Regarding the polymer DCPD-based monomers, the available information in the literature is quite scarce. Most of the information is derived from the Telene^®^ company (Bondues, France), and data sheets for this material can be found in [37,38]. However, when seeking more detailed information in the literature, it is challenging to find authors who have addressed this material and studied its mechanical properties at either low or high strain rates. Zhang et al. [39] studied the performance of the material for different ratios of monomer to catalyst. The authors present results of the tensile properties (tensile strength and elongation at-break), as well as stress–strain curves, as the ratio of monomer to catalyst is increased. According to the obtained results, an increase in this ratio implies a decrease in mechanical strength capabilities. For example, considering the n_DCPD_/n_Cat_ ratio (the ratio of the quantity of monomer and catalyst used in polymerization) of 5000:1, it was observed that the tensile modulus was 1870 MPa, and the tensile strength was 53.0 MPa, whereas for a ratio of 50,000:1, the authors obtained approximately 1500 MPa for the tensile modulus and approximately 38.0 MPa for the tensile strength.

David et al. [40] studied poly(dicyclopentadiene) (pDCPD) samples of the Telene 18xx type and conducted tensile tests at 2 mm/min on samples measuring 12 mm × 5 mm × 0.06 mm. The main objective of this study was to analyse the influence of oxidation on the mechanical properties of stabilized pDCPD at temperatures ranging from 20 °C to 120 °C. This study also analysed crack propagation, concluding that the tests showed clear indications of embrittlement of the material during oxidation, with a significant decrease in strain at break.

Another work in this area was performed by Min Yoo et al. [41], whose main objective is the manufacturing of glass-fibre/pDCPD composites with improved mechanical properties. This work provides information on the tensile, flexural, and impact strengths of the composite (with different fibre contents) and neat pDCPD (in its unmodified form). The results indicate that pDCPD when combined with glass fibre leads to increased mechanical strength. For glass fibre contents of 25%, 40%, and 55%, the tensile strength increases by 50%, 76%, and 101%, respectively, compared to the neat material (55 MPa ± 1.1).

This study represents a significant contribution to the literature by exploring the transition from glass-fibre-reinforced polymer (GFRP) to dicyclopentadiene (DCPD) in the automotive industry. This transition offers potential benefits, including lower production costs and a more environmentally friendly material. However, in the context of crashworthiness, it is crucial to assess whether this substitution maintains the vehicle’s ability to withstand impacts. A key distinction of this work focuses on DCPD, a material with limited documentation in the literature, particularly regarding its mechanical properties under intermediate and high strain rates. In contrast, while GFRP has been more extensively studied, its properties can vary significantly due to the inherent manual manufacturing process. Given the scarcity of information on DCPD and the variability in GFRP characterization, this research provides valuable and novel insights that contribute meaningfully to the existing knowledge.

## 2. Materials Manufacturing Process

The widely used hand lay-up process is employed to manufacture composites with fibre-glass reinforcement. In this process, the fibre-glass mattress is manually placed in an open mould, and resin is applied using rollers. The resin impregnates the core and cures with the help of a catalyst, creating a composite without external heat. Polyester and epoxy resins are commonly used as matrixes, curing at room temperature [42].

Among the unconventional alternatives, the reaction injection moulding (RIM) method stands out. It is based on the combination of two or more liquid components inside a mixing head, which, after being injected into a mould, gives origin to the in situ polymerization of the material [43,44]. This technology requires low-temperature processing and, because of the low-viscosity monomers/oligomers utilized, reduced injection pressures as compared to thermoplastic injection moulding (TIM) [45]. This enables the usage of a wider range of materials in the moulds, depending on the production needs, such as surface quality, production volumes, or component complexity [46]. This technology also allows for the moulding of large-sized objects with complex geometry at significantly lower pressures and clamping forces [3,38,46]. Typical parameters of RIM process can be consulted in the Table 3 [38,45,47].

Polyurethane injection has always been linked to the progress and improvement of RIM technology. However, novel polymeric materials for RIM, such as polyurea, epoxy and unsaturated polyester resins, dicyclopentadiene (DCPD), polyamides, and acrylics, have gained relevance in recent years [48]. The DCPD polymer is the result of the chemical reaction of two monomers, where no release agent or post-cure treatments are necessary [3]. It also has a Class A final surface quality [49], which is required by the automobile industry, and strong adherence to the paint [38]. This material was originally used in trucks and agricultural vehicles, competing with fibre-glass-reinforced polyester and aluminium [45].

Mixing DCPD with other materials that give structural qualities is difficult, such as metals, due to the fact that DCPD can be reactively inert to other materials, not stimulating adherence to other molecules without standard glue procedures. As a result, other complimentary processes must be used to generate hybrid polymer–metal components [48]. DCPD has environmental benefits, since the material offers low energy use and CO_2_ emissions in its production process. At end-of-life, it can be incinerated, providing substantial energy recovery from its chemical content [50]. On the other hand, DCPD is a polymer with excellent properties for applications in the automotive industry, allowing it to promote characteristics such as greater energy absorption, enhanced by the polymeric material, combined with a reduction in weight [50,51]. Indeed, recent studies describe the use of DCPD as a matrix in a glass-fibre composite that exhibited about fifty percent greater strength in impact tests and four times longer duration in the fatigue test, compared to produced samples with an epoxide matrix, maintaining the same tensile strength [52].

Since the 2000s, Telene^®^ has stood out as a leading company in RIM product manufacturing, especially its DCDP-based products. Their brochure showcases the latest technical data sheet for the Telene series, providing the detailed characteristics and mechanical properties of this material, which can be consulted in Table 4 [37,38].

**Figure 1:**
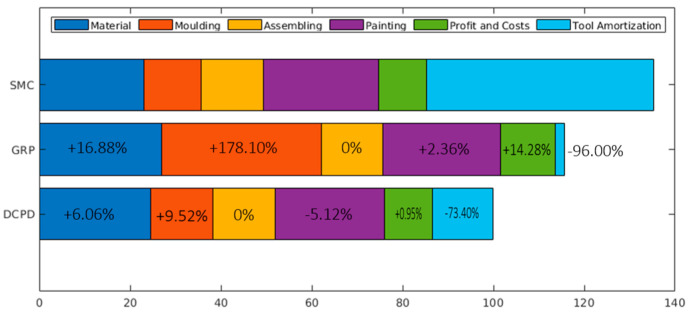
Relative costs of Telene^®^ DCPD and GRP compared with SMC for low production volumes, adapted from [48].

Regarding costs, Telene SAS conducted a comparative study of the RIM process with various other processes, including sheet moulding compounds (SMCs). For small volumes, they compared it with the hand lay-up process. Among several practical application examples where different processes can be utilized, this study concluded that the RIM production line has lower costs, as can be consulted in Figure 1 [48,53].

The production of glass-fibre-reinforced polymer involves the following steps: it features a matrix of unsaturated orthophthalic polyester resin (with a heat deflection temperature of 100 °C), reinforced by type E glass fibres randomly distributed. The properties of the cured resin are summarized in Table 5, while detailed technical data for the Ecomat 450 CNW (Emulsion) 450 gr/m^2^ glass fibre type are provided in Table 6. Next, an isophthalic polyester gelcoat finish is applied with a brush, adding a thickness of 0.5–0.8 mm to the 3 mm laminate. The production method is hand lay-up. For the curing process, post-curing is performed in the mould to prevent warping, following the resin’s recommended cycle as specified in the technical sheet. Typically, the suggested post-curing temperature for polyester resin is half an hour at 80 °C or one hour at 40–60 °C. The final colour is achieved by using a transparent resin from Gelcoat RAL 7035. The glass fibre content meets the requirements of ISO 1172:1996—Calcination, according to the manufacturer, which has now been revised and published as ISO 1172:2023 [54].

The production method of DCPD by RIM involves several steps compared to the GRP method, but DCPD production is more efficient and faster. Furthermore, in DCPD production by RIM, mould assembly and material heating are performed only once. The streamlined and continuous process of DCPD enhances its production speed and overall efficiency. The material is designated Telene^®^ 1650 A/BK. According to the safety data sheet for TELENE^©^ 16XX/26XX A, its chemical composition includes 80–90% dicyclopentadiene, 0.5–1% 1,3-dichloro-2-propanol, and 9–19.5% other undisclosed substances, [57]. The resins (components A and B) are stored commercially in nitrogen-blanketed steel drums, with the injection system being automated and operated at low pressure. These resins are processed using RIM equipment, maintaining a fixed 1 to 1 A/B ratio. An independent pump system transfers the components from separate units and injects them into the mix head after filtration. After a turbulent mixing process, the resin is injected into the mould in a laminar flow to prevent defects [58]. The curing process occurs within the mould and is highly exothermic. Consequently, the heating and cooling lines are designed to maintain the mould surface temperature within ±2 °C of the set point. Lastly, finishing procedures such as trimming, surface preparation, cleaning, painting, and storage are performed. Further information can be found in the Refs. [58,59].

## 3. Test Methodology

Quasi-static and dynamic tensile tests were conducted employing multiple pieces of equipment. The tests were performed on each material in line with ASTM D3039 [60] and ASTM D638 [61], and the experiments referenced describe the “standard tension”. In order to overcome the experimental limitations, “non-standard tension” geometry was adopted. The nominal dimensions and non-standard configurations of the specimens for both materials are shown in Table 7 and Table 8. A summary of the performed tests is presented:

- Quasi-Static (QS): tested using a custom Portable Testing Machine (PTM);
- Intermediate Strain Rate: 1 s^−1^ and 10 s^−1^ tested using a high-speed machine (HSM);
- High Strain Rate: 150 and 250 s^−1^ tested using a split Hopkinson pressure bar (SHPB) apparatus.

**Figure 2:**
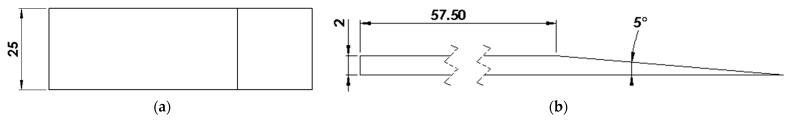
End tab drawing for GFRP tensile testing, in [mm]: (**a**) top view and (**b**) front view.

The polymer material is reinforced with fibre glass and random-discontinuous fibre orientation. Therefore, end tabs were used to prevent gripping damage during standard testing. Figure 2 shows a depiction of the end tab of aluminium alloy material. There was no slippage between the grips and the specimen while using a mechanical clamping mechanism with a tightening force of 3.5 kg.m (34 N.m). The created fixture was integrated into the testing machine in accordance with the non-standard testing typology.

The rectangular specimens were attached to two slotted tab adapters using Araldite 2014-2 adhesive, which has a shear strength of 30 MPa, tensile modulus of 3100 MPa, and elongation at break of 0.9%. To ensure proper bonding, a fixture was used to maintain alignment between the specimen and steel adapters during the adhesive curing process, following the supplier’s recommendation of 24 h at 23 °C. Furthermore, mechanical clamps were applied to offer additional support, preventing any in-plane misalignment of the sample during testing. The equivalent density of the material was obtained from experimental measurements of the specimen geometry and mass, so the density of DCPD is 1.313 g/cm^3^ and 1.665 g/cm^3^ for GFRP.

### Test Setup Characterization

**Figure 3:**
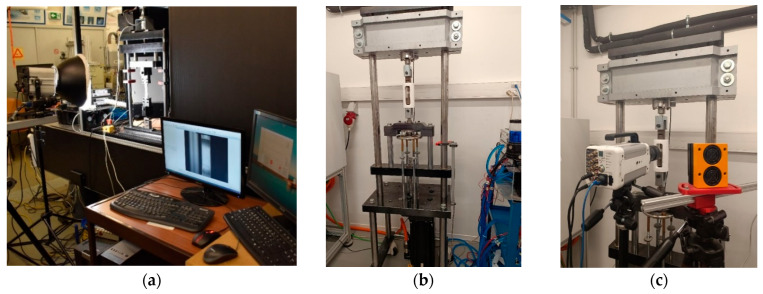
(**a**) A view of the quasi-static test setup equipped with 2D-DIC and spotlight, (**b**) a view of the HSM apparatus, and (**c**) HSM testing machine equipped with 2D-DIC.

Figure 3a provides a view of tensile testing for QS tests outfitted with a DIC data collection device, to acquire the full-field strain contour of all trials. The tests were performed with a constant crosshead speed of 2 mm/min and 5 mm/min for the GFRP and DCPD, respectively. The shots were captured using a Point Grey Gazelle GZL-CL-41C6M-C 4.1MP (Point Grey, Richmond, Canada)-equipped Rodagon 1:4 lens (Rodenstock GmbH, Munich, Germany) and lighted by a Hedler DF15 150 W light unit (Hedler Systemlicht GmbH, Runkel, Germany). Based on the area of interest, sensor size, free work distance, and camera mount, an optimum lens combination was used. VIC-2D was used to post-process the pictures [62].

This equipment was developed in-house for static or quasi-static testing, in which further information can be found in Ref. [63]. The motor is a QBL4208-100-04-025 BLDC from Trinamic (Hamburg, Germany), coupled with a 1:70 reduction gearbox, and the load cell model 101BH-SE2t is supplied by Vetek (Väddö, Sweden), along with an encoder model GVS 206 S [63]. Table 9 presents the optical strain measurement parameters for both test setups during the acquisition and post-processing stages.

Intermediate strain rate tests were carried out on a high-speed testing machine, developed at INEGI (as shown in Figure 3b,c), supporting tensile forces up to 50 kN at constant speeds ranging from 1 mm/s to 1100 mm/s. After placing the specimen in the gripping assembly, the downward movement of the impactor is activated by a low-inertia brushless servo motor (model MPL − B680H − MJ74AA from Rockwell Automation (Milwaukee, WI, USA), [64]). The proper distance between the impactor and the plate, at the beginning of the test, allows the acceleration of the impactor up to the defined test velocity, ensuring a constant rate. The force applied is measured by a 50 kN dynamic load cell (model TC4 50 kN from Vetek, [65]), placed between the upper grip and the fixed crossbeam of the machine, at an acquisition rate of 750 kHz. The position of the impactor is recorded by a linear encoder attached to the base of the impactor at an acquisition rate of 40 kHz.

DIC technique is employed to monitor the local strain field. The pictures are taken using a FASTCAM Nova S6 high-speed camera (Photron, San Diego, CA, USA) and a Nikon 60 mm micro lens with a resolution of 1024 × 1024 pixels (Tokyo, Japan). To light the specimen’s surface, a MultiLed MX High Power LED (Photo-Sonics International Ltd. Thame, UK) [66] was used. Table 10 summarizes the acquisition settings. The records of the applied force, test specimen deformation, and impactor position are synchronized through a 5 V electrical pulse with a duration of 100 ms emitted by the camera and transmitted to the other recording equipment.

Five tests per strain rate per material were conducted using the HSM. After several trials using the values recorded by the encoder, the test velocities ranged from 50 mm/s to 90 mm/s for the lower strain rate and from 420 mm/s to 585 mm/s for the higher strain rate, resulting in strain rates of 1 s^−1^ and 10 s^−1^, respectively.

**Figure 4:**
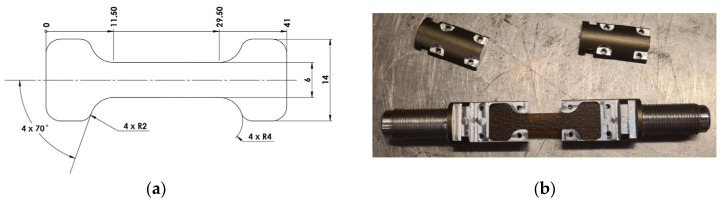
The miniaturized specimens: (**a**) 10 mm gauge length (2D dimensions) and (**b**) and gripping system.

To perform these tests, it was necessary to reformulate the geometry of the specimens. Currently, there are no standards that stipulate a standardized geometry for strain rates beyond the scope of typical strain rates observed in QS tests. According to Smerd et al. [67], it is necessary for the specimen to have a small gauge length in order to reduce the ring-up time (referring to the time it takes for the stress wave to travel back and forth in the specimen) and inertial effects [68,69]. On the other hand, to ensure that the specimen fractures in the middle section, a dog-bone-type geometry was adopted, where the central section is narrower than the area where the specimen is mounted. Regarding the geometry, it is also compatible with a dynamic test setup, similar to the one proposed in Shamchi’s study [68]. This configuration can be seen in Figure 4. Regarding to the post-processing, in order to filter the signal, a moving average filter with 500 samples was employed.

High strain rate mechanical characterization of both supplied materials was carried out in tension using the SHPB setup. The specimens were manufactured following the same specifications as previously presented in Figure 4. The SHPB apparatus, also commonly referred to as the Kolsky bar, is one of the most widely used methods for studying materials with strain rates in the range of 10^2^ s^−1^ to 10^4^ s^−1^, and it was pioneered by John Hopkinson [68,70,71]. Throughout history, it has been noteworthy to highlight the system presented by Bertram Hopkinson, Davies, and Kolsky, who continuously sought to improve the original system. Subsequently, Harding J., Wood, E. D., and Campbell, J. D. extended the technique to tensile tests, which until then had only been used for compression tests. The use of strain gauges to measure displacement was introduced by Hauser et al. [70].

This system typically consists of two solid bars (made of homogeneous and isotropic material) of equal diameter and uniform cross-section, a pressurized air chamber (usually the loading mechanism), and a data acquisition system [29,72]. The bars must be sufficiently long to provide interference-free propagation of the waves, enabling the subsequent acquisition of the stress–strain curve [72]. For the tensile test, the pressure reservoir is pressurized, and upon triggering, the striker bar impacts the incident bar at a velocity of v_0_, generating a compression wave, later transformed into the incident pulse, ε_i_, with approximately rectangular geometry [29,72]. This wave is measured using a strain gauge positioned on the incident bar. When the incident wave passes through the specimen, a percentage is reflected backward as a compression stress pulse (due to the difference in cross-section), while another part is transmitted to the specimen, which in turn transmits a wave to the transmitted bar, known as the transmitted pulse, ε_t_, and measured by the strain gauge positioned on this transmitted bar [68]. Understanding the strain waves is crucial for calculating the dynamic response of the specimen [72].

**Figure 5:**
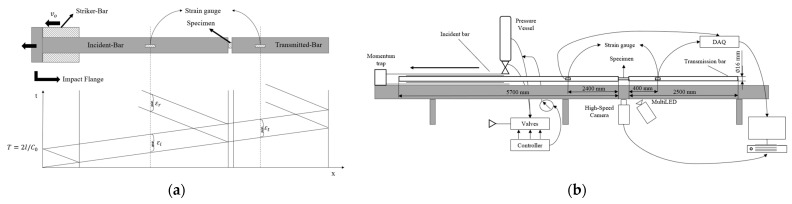
(**a**) Schematic of Lagrangian diagram for tensile setup impact wave propagation, in SHPB apparatus, adapted from Ref. [68], and (**b**) schematic of the split Hopkinson pressure bar assembly, based on Sousa et al. [75] and Shamchi et al. [73].

According to Ref. [68], this model is designated as a 1D stress wave propagation, and the wave propagation model can be illustrated through the Lagrange diagram, as depicted in Figure 5a. The pulse duration, T, represented in the Lagrange diagram depends on the length of the striker bar, l, and the elastic wave velocity of the bar material, c_o_, [29,68]. The strain gauges will allow the stress–strain curve of the deformed sample to be obtained using the conventional equations of the Hopkinson bar [73]. Currently, the available Hopkinson bar system for testing materials consists of a custom-built pneumatic setup, comprising two compression and two tension bars [73,74]. This system requires manual activation of multiple acquisition systems, including pressurization of the reservoir and firing, as well as the acquisition of stress signals for both bars (incident and transmitted), by employing strain gauges complemented by full-field strain measurements using DIC. A schematic of the tensile setup bar can be seen in Figure 5b.

The bars that comprise the equipment are constructed of titanium alloy (Titanium Gr. 5) with a yield strength of 275 MPa and an elastic modulus of 105 GPa. The incident bar has a length of 5700 mm, while the transmitted bar has a length of 2500 mm, both with a diameter of 16 mm. Regarding the positioning of the strain gauges, the incident bar is positioned 2400 mm from the specimen’s edge, while the transmitted bar is positioned 400 mm from the specimen’s edge. The strain gauges used in the system have the reference CEA-06-125UN-120, being supplied by Micro Measurements company (Wendell, NC, USA). These strain gauges have a resistance of 120 Ω and a gauge factor of 1.98, in which it is suitable for dynamic applications, allowing the use of higher excitation voltages and providing a larger output signal [72]. Next, the acquired data from the initial samples were recorded and analysed using an open-source software, SUREPulse 1.23.0. Additionally, the pulse duration is about 620 μs. The experimental data acquisition and signal processing system comprise six-channel Pico PC Oscilloscopes (Pico Technology, St Neots, UK) equipped with software resolution enhancement to 16 bits and a 23MS buffer memory.

All experiments are supported by data used for DIC obtained using a high-speed camera, the Photron^®^ FASTCAM NOVA model S6 800K-M-16GB. The camera was positioned so that the deformation applied to the specimen during testing was fully captured in detail, to be subsequently employed in post-processing. A MultiLed MX High Power LED was used to illuminate the specimen’s surface. Regarding the optical parameters used in these tests, they may be consulted in Table 11. Regarding post-processing, in order to filter the signal, the load data were filtered for 4 kHz, and the displacement data were filtered for 10 kHz. In these experimental tests, it was ensured that the stress equilibrium was achieved by comparing both the input and output bar forces. Also, the wave dispersion was taken into account, so it was decided to follow Shamchi’s studies [68]. According to Ref. [76], the authors used a pulse shaper made of copper wire at the end of the incident bar for impact absorption, attenuating the wave dispersion along the bar.

## 4. Results

### 4.1. Quasi-Static Results

**Figure 6:**
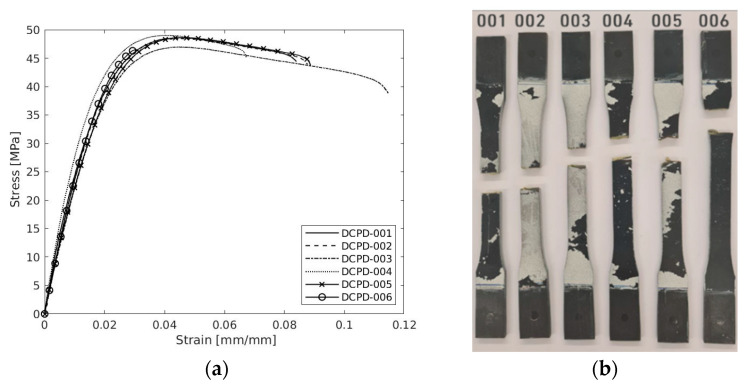
(**a**) Tensile properties of the DCPD polymer under the quasi-static loading and (**b**) failure mode of the specimens.

Regarding the DCPD material, Figure 6a presents the quasi-static (QS) results behaviour for all tested specimens. All six of the performed tests were repeatable, demonstrating a linear stress–strain relation up to the yield stress, which corresponds to the point where the curves end the linear behaviour. The strength for all specimens demonstrates the huge similarity as comprised by the low variance. On other hand, there is some variation concerning the failure strain, which corresponds the endpoint of the curves.

All the QS specimens exhibited a failure mode in the form of a transverse direction to the loading direction. The failure mode pictures are shown in Figure 6b. Once the maximum load was reached, the samples failed abruptly within the gauge section, with the exception of specimen 006, which failed nearest to the clipping area at low strain compared to the other samples. This last-mentioned sample cannot be validated with further calculations.

Mean values of 2053 MPa, 48.56 MPa, and 8.82% for elastic modulus, ultimate strength, and failure strain, respectively, were measured with a coefficient of variance of 5.96%, 1.11%, and 19.09%, respectively. It is observed that the strength has a low coefficient of variance and a higher value for failure strain. The findings from the experiments are summarized in Table 12.

**Figure 7:**
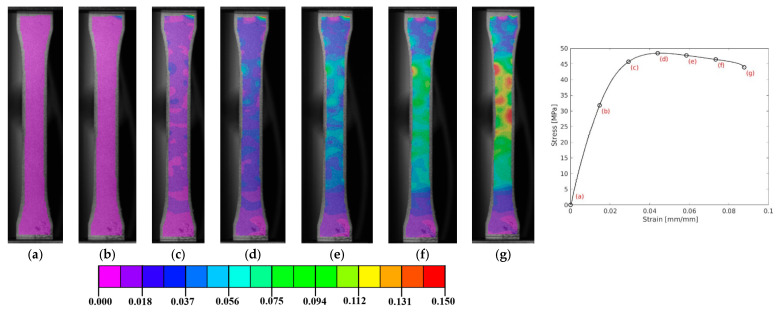
A representative axial strain field distribution of DCPD polymer (**a**–**g**)—specimen 002, for quasi-static testing.

Regarding the DIC results, a representative axial strain field is presented in Figure 7. It is observed that the strain distribution is quite uniform until reaching ultimate strength. Furthermore, this material is made through the injection of different compounds into a matrix. This factor can cause non-uniform material properties in several areas due to the temperature, which may cause failure in distinct regions, which may explain why this measurement has a higher coefficient of variation. On the other hand, this material presents a ductile fracture due the presence of a large plastic regime [77].

A detailed observation of specimen 002 for DCPD, shown in Figure 7, reveals that, despite the relatively uniform distribution of damage (manifested as “islands”, the higher-strain areas), the concentration of damage occurs primarily at the narrower section of the specimen, as expected. From step (d) to (f), leading up to the final failure in (g), the maximum strain is confined to this region of the specimen. This localized strain accumulation contributes to the progressive degradation of the material’s strength, as illustrated in the enclosed graphic. This behaviour is consistent across the other specimens shown in Figure 6b.

**Figure 8:**
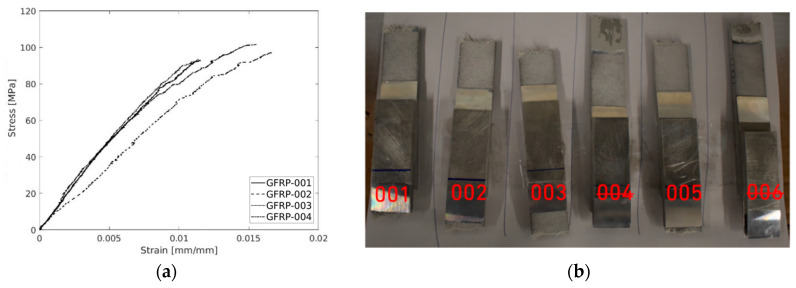
(**a**) Stress–strain plots of the GFRP and (**b**) failure mode of the specimens.

Figure 8a depicts the behaviour of the stress–strain plots of the tested specimens, which were mainly successful. The tests were carried out, indicating a repeatable strain relationship up to the yield stress, with failure occurring without entering the plastic regime, which is according to the results found in the literature. The test also shows a high degree of resemblance to all tests excluding specimen 004, which was considered invalid since fractures inside the grips occurred. Table 13 displays the values in further detail.

It is possible to observe failure in mode I (tensile opening), mode II (in-plane shear), or mode III (out-of-plane shear) [78]. In this test, mode I is predominantly expected. However, due to the nature of the material, mode II will also occur when delamination (non-uniform inter-layer failure or progressive layer damage propagation) is observed, particularly between layers of the laminate. Past the peek stress point, the test specimens exhibited a “burst” failure mode. However, to improve the clarity of the graphs, the final part of the test was removed from the data, thereby hiding the sudden load breakdown.

The experimental measurements yielded mean values of 9370 MPa, 96.29 MPa, and 1.37% for the elastic modulus, ultimate strength, and failure strain, respectively. These measurements were associated with coefficient of variance values of 18.39%, 4.21%, and 19.84%, respectively. Notably, the ultimate strength exhibited a low coefficient of variance, indicating a relatively consistent performance, while failure strain and elastic modules showed a higher variability. The findings from the experiments are summarized in Table 13.

**Figure 9:**
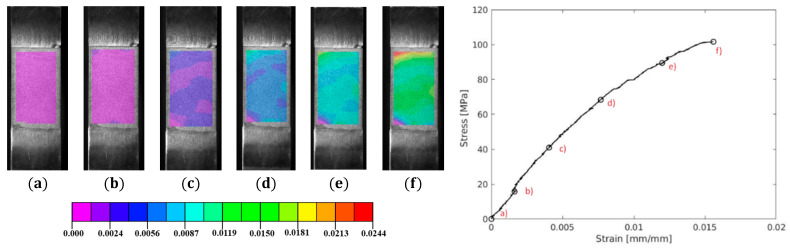
A representative axial strain field distribution of GRFP—specimen 005 (**a–f**), for quasi-static testing.

In Figure 9, a representative axial strain field provided by DIC analyses is presented for specimen 005. The visualization reveals a relatively uniform strain distribution until reaching ultimate strength. Notably, the area near the boundary between the tab and the specimen showed potential stress concentrations that could lead to premature failure [79]. This variability might contribute to failure in this specific region, offering an explanation for the higher coefficient of variation observed in these measurements.

Additionally, as shown in Figure 8a, even the best results exhibit a high degree of uncertainty (using end tabs with 5°), as subsequently presented in Table 13. The stress –strain curves reveal irregular behaviour. In order to try to improve the results, several tests were conducted using tabs manufactured from the same material (aluminium alloy material) but for 90° instead 5° (see Figure 2). However, the results were worse, as this angle introduced more localized residual stress near the clamping area. In general, the tests for 90° end tabs were unsuccessful due to adhesive–probe bond failure or fracture occurring within the grip, rendering most of the results invalid.

An additional trial involved employing specimens with a dog-bone geometry, as expressed in Table 14. The width of the specimen was decreased by 8 mm to lower the necessary ultimate load for a valid failure, ensuring a representative volume of the GFRP. Dog-bone-shaped specimens have proven successful in the context of GFRP [80].

**Figure 10:**
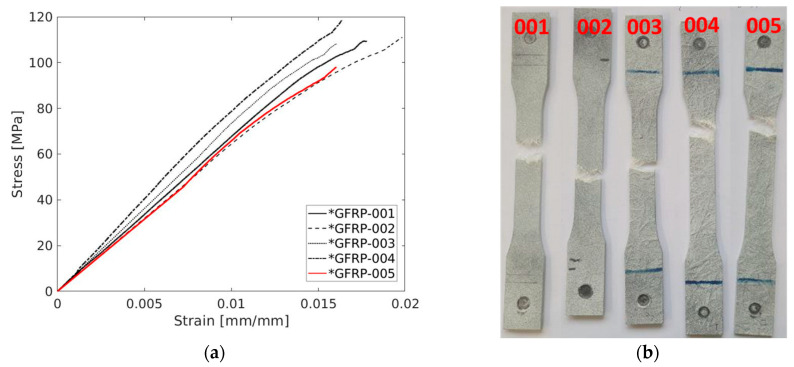
(**a**) Stress–strain plot of the glass-fibre/polyester polymer and (**b**) failure mode of the specimen’s dog-bone geometry.

Regarding the results of these trials, Figure 10a illustrates the QS stress–strain behaviour for all tested specimens. Each of the five conducted tests demonstrated a high degree of repeatability, showing a more linear stress–strain relationship up to the yield stress. The strength exhibited remarkable similarity among all specimens, resulting in low variance. However, there was some variation in terms of failure strain, representing the endpoint of the curves, although lower compared to the previous tests. All quasi-static specimens displayed a failure mode in the transverse direction of the loading axis. The pictures depicting the failure mode are presented in Figure 10b.

**Figure 11:**
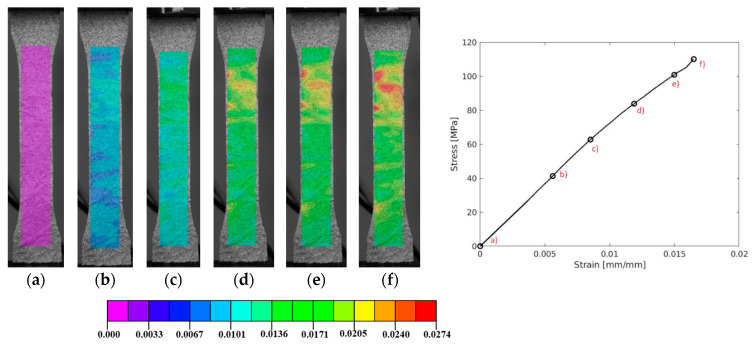
A representative axial strain field distribution of GRFP—specimen 004 (**a**–**f**), for quasi-static testing for non-standard geometry.

The experimental measurements yielded mean values of 6774 MPa, 106.56 MPa, and 1.74% for the elastic modulus, ultimate strength, and failure strain, respectively. These values were associated with coefficient of variance values of 5.69%, 5.52%, and 10.40%, respectively. Remarkably, the coefficient of variance associated with each parameter was reduced with this geometry, proving to be more successful. A summary of the experimental findings is presented in Table 15. In Figure 11a, a representative axial strain field provided by DIC analyses is presented for specimen 004.

### 4.2. Dynamic Results—Intermediate Strain Rate

Attempts were made to fix the strain rate at the same value for both materials, at about 1 s^−1^ and 10 s^−1^. For this purpose, two sets of tests were conducted, comprising five specimens each. Thus, for the first round of tests (first set of tests), on DCPD, an average of 1.12 s^−1^ was observed with a coefficient of variance (CV) of 11.68%, while in the second round (second set of tests), 9.95 s^−1^ was obtained with a CV of 7.21%. In addition, regarding GRFP, the first round of tests revealed an average of 1.05 s^−1^ with a CV of 31.97%, whereas in the second round, the values were 9.67 s^−1^ and CV 10.01% respectively. The strain rate was obtained by calculating the slope of the time–strain curve of each test by employing a virtual extensometer.

**Figure A1:**
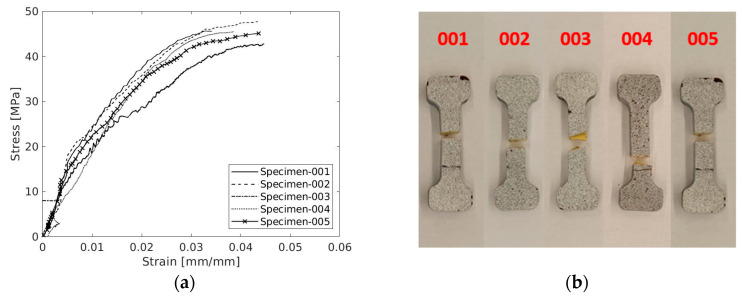
(**a**) Tensile properties of the DCPD polymer for 1st set of tests under intermediate strain rate loading and (**b**) failure mode of the specimens.

Starting with DCPD, Figure A1a depicts the stress–strain behaviour for the first set of results. All five tests conducted show repeatability in the results, demonstrating an approximately linear stress–strain relationship up to the yield point (CV of 8.34%), which corresponds to where the curves depart from linear behaviour. The ultimate strength of all samples demonstrates significant similarity with low variance (3.97%). However, there is higher variation in the failure strain (10.95%), which corresponds to the endpoint of the curves.

All tested samples exhibit a failure mode in the form of a transverse direction to the loading direction. The failure modes are shown in Figure A1b). Once the maximum load is reached, the samples can withstand the load with a slight decrease in stress until fracture. In this set of tests, specimen 003 stands out, reaching the maximum stress (42.81 MPa), and slightly higher elongation is observed (failure strain = 4.46%). This latter-mentioned sample causes an increase in the standard deviation of the values, as verified. Experimental measurements produced average values of 1985 MPa, 45.38 MPa, and 4.09% for the modulus of elasticity, ultimate strength, and rupture strain, respectively. The experimental findings are summarized in Table 16.

**Figure A2:**
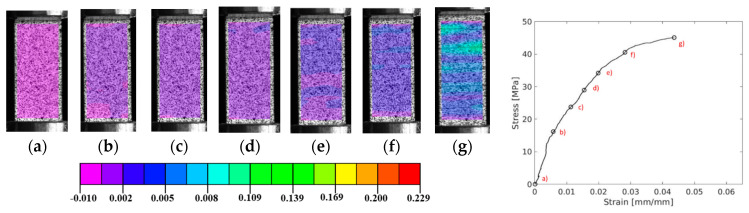
A representative axial strain field distribution of DCPD polymer—specimen 005 (**a**–**g**), for 1st set of tests of intermediate strain rate.

In Figure A2, the case of specimen 005 is presented in detail by analysing the representative strain field in the orientation of the loading direction extracted from VIC. The sequence of images follows the chronology represented by the nomenclature shown in the stress–strain curve in the same figure. From the initial loading stage, it is observed that the distribution of loading direction strains is homogeneous, although at moment (e) slight transverse fractures are observed on the surface, becoming more pronounced until fracture at (g). This behaviour is slightly different from what was observed in the QS tests, visible in Figure 7. In the QS tests, these surface fractures are not strictly visible, meaning that the material can accommodate the loading state at low strain rates.

**Figure A3:**
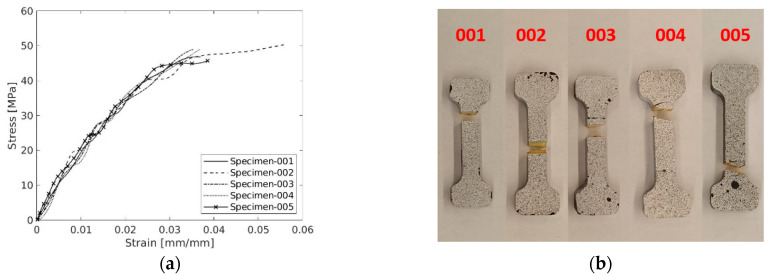
(**a**) Tensile properties of the DCPD polymer for 2nd set of tests under intermediate strain rate loading and (**b**) failure mode of the specimens.

On the other hand, Figure A3 depicts the stress–strain curves for the second set of results, as well as the failure mode. Failure occurs identically to the previously conducted tests, in the transverse direction to the loading direction. All five conducted tests demonstrated high repeatability in the results; however, the behaviour differs from the 1 s^−1^ and QS strain rates. For this strain rate, the behaviour tends to differ, as can be seen in Figure A3a, where the shape of the curves is significantly affected by the appearance of oscillations. In Ref. [81], the author states that for strain rates on the order of 8.7 s^−1^, the appearance of oscillations due to impact-like loading began, which is close to 10 s^−1^ [81].

The experimentally measured results are summarized in Table 17. Average values of 1977 MPa, 47.69 MPa, and 4.07% were determined for the elastic modulus, ultimate stress, and failure strain, respectively. Regarding the coefficients of variation, the following values were measured: 7.35%, 3.63%, and 20.94%, respectively. In this case, there is greater variation in failure strain (20.94%), due to the elongation of specimen 002.

**Figure A4:**
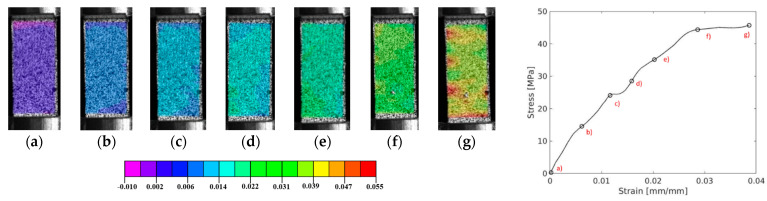
A representative axial strain field distribution of DCPD polymer—specimen 005 (**a**–**g**), for 2nd set of tests of intermediate strain rate.

The strain contour field in the loading direction for specimen 005 is presented in detail in Figure A4. From the initial loading stage, seven loading states are represented. With the dynamic loading, it is observed that the distribution of vertical axial strain (loading direction) is reasonably homogeneous. However, unlike the 1 s^−1^ loading, the transverse fractures are only more visible in the state very close to fracture, as indicated by the strain closer to the failure limit represented by the red colour in the contour field. Therefore, with the increase in strain rate from 1 s^−1^ to 10 s^−1^, the behaviour of the multiple transverse micro-superficial cracks tended to decrease, i.e., to be less visible. Another aspect is that the value of the failure strain reduced by approximately 0.02% (average values), i.e., the failure strain is not affected by the strain rate.

**Figure A5:**
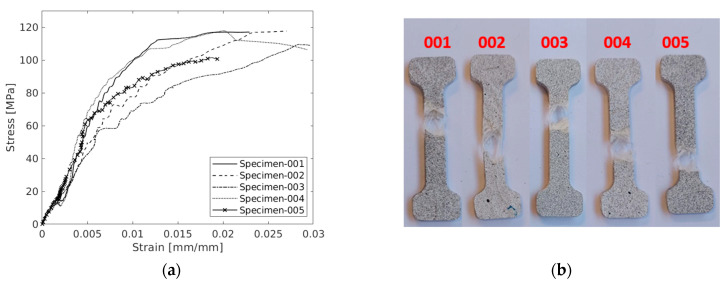
(**a**) Tensile properties of the GRFP for 1st set of tests under intermediate strain rate and (**b**) failure mode of the specimens.

Regarding GRFP, the same loading conditions are applied as previously. Figure A5a presents the stress–strain curves for the first set of results, at an intermediate strain rate, for all tested samples, as well as the failure mode (Figure A5b). The experimentally measured results are summarized in Table 18. Average values of 10,158 MPa, 112.70 MPa, and 2.56% were determined for the elastic modulus, ultimate stress, and failure strain, respectively. Regarding the coefficients of variation, the following values were measured: 12.70%, 6.38%, and 17.31%, respectively. In this specific case, it is observed that the coefficients are relatively higher than those for DCPD, possibly due to the manual manufacturing process introducing a higher degree of uncertainty in the repeatability of the results, unlike DCPD, which comes from an automated process.

Concerning the fracture behaviour of GRFP at this strain rate, it is observed that fractures exhibit rougher and more irregular edges on the surface, where matrix cracks, fibre breakage, and fibre pull-out occur. The failure mode of GRFP specimens is presented in Figure A5a. It is possible to compare them with the results obtained in the QS tests. At QS, the fracture surface is confined to a small area where fibre breakage is significantly reduced, and irregularity is lower. However, with increasing strain rate, the damage mode tends to cover a larger section of the specimen. Extensive fibre–matrix debonding was observed, leading to an increase in tensile strength and energy absorption [82].

**Figure A6:**
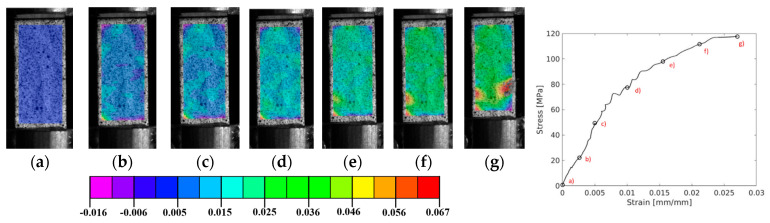
A representative axial strain field distribution of GRFP—specimen 002 (**a**–**g**), for intermediate strain rate (1st set of tests).

Figure A6, presents specimen 002 in detail, where it is possible to observe the full-contour strain field in the loading direction, in which seven loading states are represented. Through the DIC analysis of the longitudinal strain field of the specimen, the initiation of a matrix crack is revealed around state (e), accentuating in state (f). The crack which leads to failure starts precisely at the opposite edge of the specimen, approximately on the same detected fault line at state (e). Furthermore, contrary the DCPD, a higher degree of stress concentration near the clamping zone was detected in this material, which induces damage under the out-of-plane clamping force. This can manifest as minor cracks and an observed non-homogeneous strain field, especially near the clamping area.

**Figure A7:**
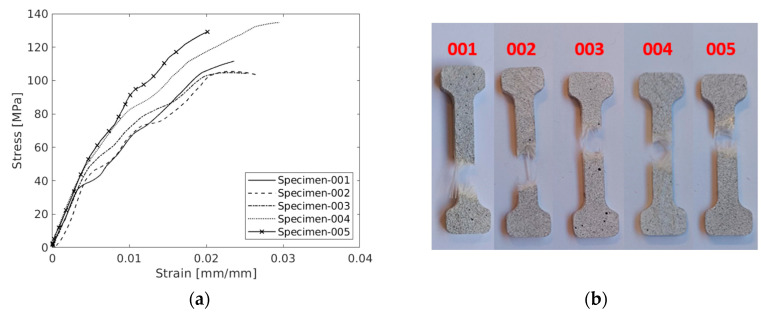
(**a**) Tensile properties of the GRFP for 2nd set of tests under intermediate strain rate loading and (**b**) failure mode of the specimens.

**Figure A8:**
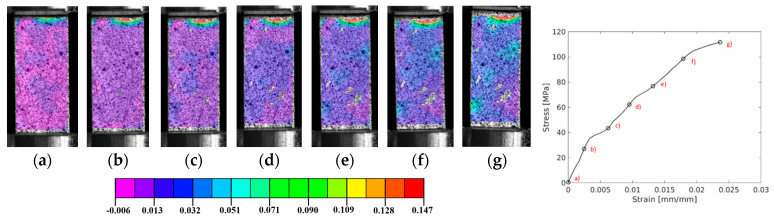
A representative axial strain field distribution of GRFP—specimen 001 (**a**–**g**), for 2nd set of tests of intermediate strain rate.

In the second set of results for GRFP, the rates are about 10 s^−1^, the following results are discussed. Figure A7a presents the stress–strain curves, and the failure modes of all tested samples are shown in Figure A7b. The experimental measured results are listed in Table 19. Average values of 10,380 MPa, 117.74 MPa, and 2.50% were determined for the elastic modulus, ultimate stress, and failure strain, respectively. For the average values found, the coefficients of variation (which express the variability of the value in relation to the mean in terms of percentage) were measured as follows: 9.00%, 11.37%, and 13.91%, respectively. In this case, it is observed that the coefficients of variation are generally lower when compared to the 1 s^−1^ strain rate, indicating that the increase in strain rate influences the repeatability of the results. On the other hand, the increase in strain rate had little influence on the changes in the average values of the properties. Figure A8 details the contour strain field in the loading direction for specimen 001, highlighting six loading states. Through the DIC analysis of the longitudinal strain field of the specimen, the uniformity of strain values in the studied field is evident, although it may be a consequence of stress concentration near the clamp. Clearly, the relative increase of 10 s^−1^ in the strain rate caused a considerable increase in stresses near the clamp, a behaviour previously observed but on a smaller scale. Subsequently, in state (f), it is again observed that the onset of fracture begins on the side, where a slight increase in strain is noted. Later, aggravation of this phenomenon leads to crack initiation, which causes brittle fracture due to the nature of the material. The brittle fracture directly affects the fracture failure mode, evidenced by the significantly higher fibre pull-out than matrix fracture, and fibre breakage is visible by the fibre pull-out.

### 4.3. Dynamic Results—High Strain Rate

Similar to what was performed previously, five attempts to obtain the strain rate with low variance are presented, around 140 s^−1^ and 262 s^−1^ for neat DCPD and approximately 128 s^−1^ and 252 s^−1^ for GRFP. For this purpose, two sets of tests were conducted. Thus, for the first round of tests (first set of tests), in DCPD, an average of 140.47 s^−1^ was observed with a CV of 4.27%, while in the second round (second set of tests), 261.53 s^−1^ was obtained with a CV of 2.67%. Regarding GRFP, the first set of tests resulted in an average of 127.54 s^−1^ with a CV of 8.83%, while in the second set, the values were 251.70 s^−1^ and CV 10.50%, respectively.

**Figure A9:**
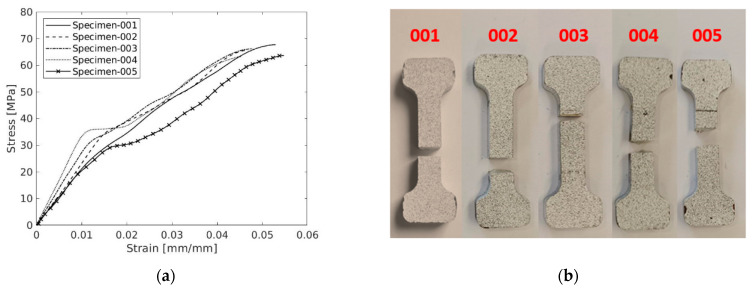
(**a**) Tensile properties of the DCPD polymer under high strain rate loading (1st set of tests) and (**b**) failure mode of the specimens.

**Figure A10:**
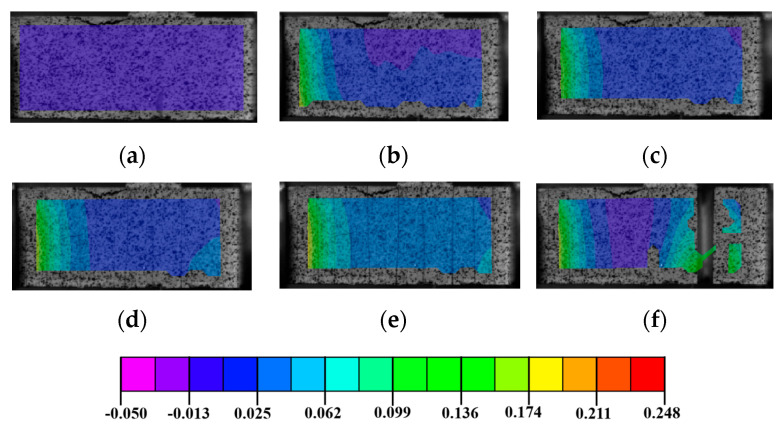
In-plane strain field distribution for DCPD polymer—specimen 001, for high strain rate (1st set of tests), for (**a**) 0 μs, (**b**) 70 μs, (**c**) 140 μs, (**d**) 210 μs, (**e**) 280 μs, and (**f**) 440 μs.

Figure A9 presents the stress–strain curves for the first set of results (140 s^−1^) for all tested samples, as well as the failure mode of the samples. The experimental results are summarized in Table 20. Average values of 2482 MPa, 65.30 MPa, and 4.95% were determined for the elastic modulus, ultimate stress, and failure strain, respectively. Regarding the coefficients of variation, the following values were achieved: 23.78%, 2.84%, and 8.24%, respectively. It is evident that for this higher strain rate, the behaviour of multiple transverse cracks tended to be more visible, possibly as a result of polymer chain rupture, as visible in Figure A10 [83]. In Figure A10, the contour strain field in the loading direction of specimen 001 is presented in detail, for the test duration.

**Figure A11:**
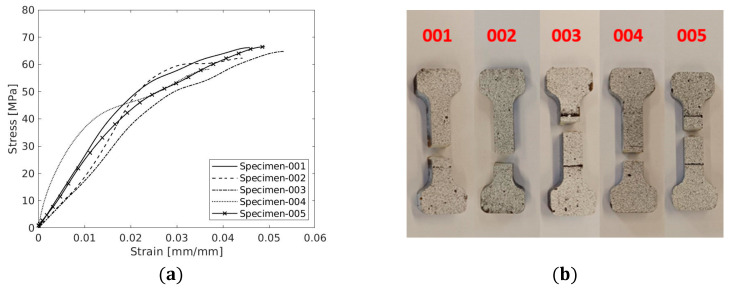
(**a**) Tensile properties of the DCPD polymer under high strain rate loading (2nd set of tests) and (**b**) failure mode of the specimens.

**Figure A12:**
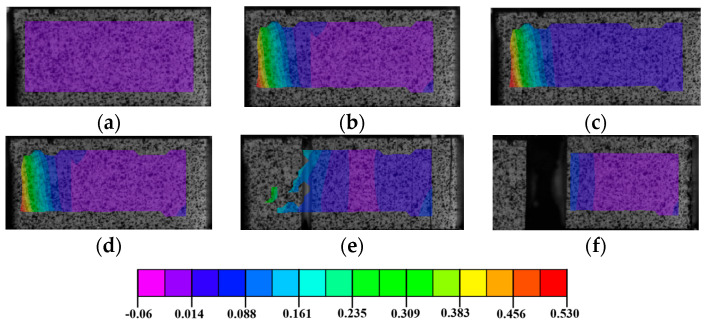
In-plane strain field distribution dynamically loaded for DCPD polymer—specimen 005, for high strain rate (2nd set of tests), for (**a**) 0 μs, (**b**) 70 μs, (**c**) 140 μs, (**d**) 230 μs, (**e**) 230 μs, and (**f**) 330 μs.

Figure A11 presents the stress–strain curves at a high strain rate (second set of results, 261 s^−1^), as well as the failure mode of all tested samples. The measured results are summarized in Table 21. Figure A12 presents the case of specimen 005 to present the contour strain field obtained by DIC.

**Figure A13:**
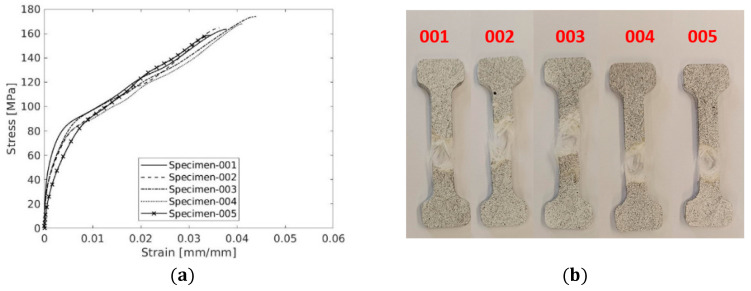
(**a**) Tensile properties of the GRFP under high strain rate loading (1st set of tests) and (**b**) failure mode of the specimens.

**Figure A14:**
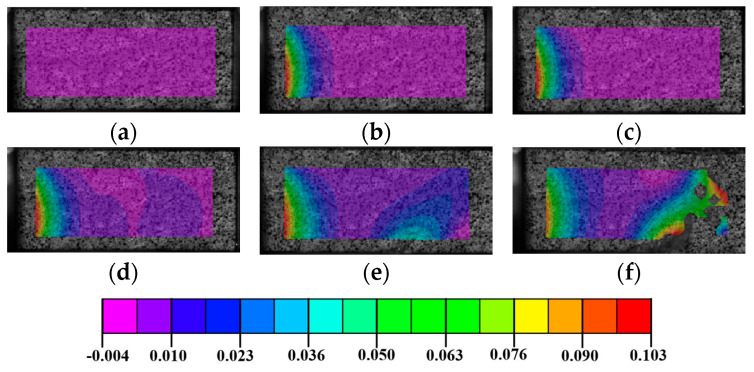
In-plane strain field distribution dynamically loaded for GRFP—specimen 001, for high strain rate (1st set of tests), for (**a**) 0 μs, (**b**) 70 μs, (**c**) 140 μs, (**d**) 220 μs, (**e**) 280 μs, and (**f**) 440 μs.

Regarding GRFP, Figure A13a illustrates the stress–strain curves obtained at a high strain rate (first set of results, 128 s^−1^) for all tested samples, along with the failure mode depicted in Figure A13b. In this specific case, it is observed that the behaviour differs from what has been previously observed, evidencing no initial linearity associated with the material’s elasticity, for this strain rate. Therefore, Young’s modulus cannot be calculated using Hooke’s law. According to Ref. [84], depending on the test speed, if it is high, it is possible to obtain a secant modulus of elasticity to approximate this behaviour. The experimental findings are summarized in Table 22, showing average values of 4299 MPa, 165.70 MPa, and 3.87% for the secant modulus, ultimate stress, and failure strain, respectively. Coefficients of variation were also calculated, resulting in 6.50%, 3.40%, and 9.83% for the respective parameters. In Figure A14, the strain field obtained is presented, focusing on the specific case of specimen 001.

**Figure A15:**
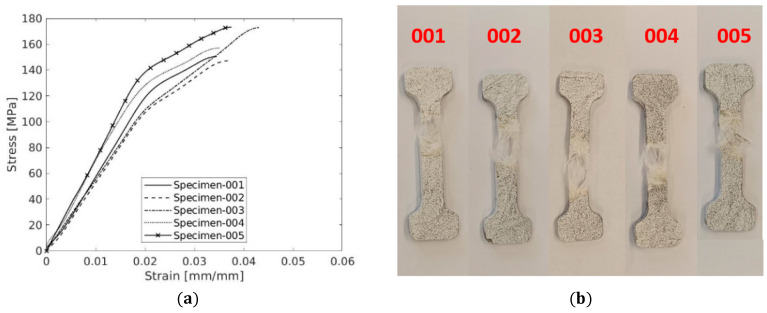
(**a**) Tensile properties of the GRFP under intermediate strain rate loading (2nd set of tests) and (**b**) failure mode of the specimens.

**Figure A16:**
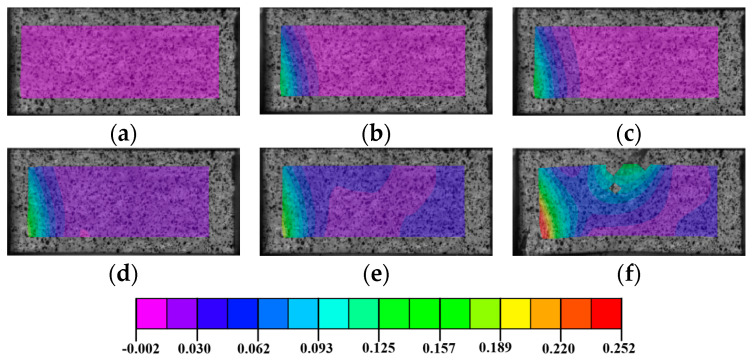
In-plane strain field distribution dynamically loaded for GRFP—specimen 005, for high strain rate (1st set of tests), for (**a**) 0 μs, (**b**) 50 μs, (**c**) 100 μs, (**d**) 150 μs, (**e**) 200 μs, and (**f**) 240 μs.

Figure A15 illustrates the stress–strain curves obtained for all examined samples, with the strain rate averaging 252 s^−1^, along with the failure mode. The experimental findings are summarized in Table 23. In Figure A16, the obtained strain field is presented, focusing specifically on specimen 005. For these higher strain rates, DIC analysis of the longitudinal strain field of the sample reveals a stress concentration near the clamping zone resulting from the impact of the striker bar. The fracture closely resembles what was reported earlier, with the emergence of a matrix crack detected on the side of the specimen, centrally in this case, visible in state (f). At this order of magnitude of strain rates, the fracture extends over a larger damaged area where fibre breakage is significantly high, stemming from the elevated energy release. Extensive fibre–matrix debonding was also observed, leading to an increase in tensile strength and energy absorption.

### 4.4. Discussion of Results

**Figure 12:**
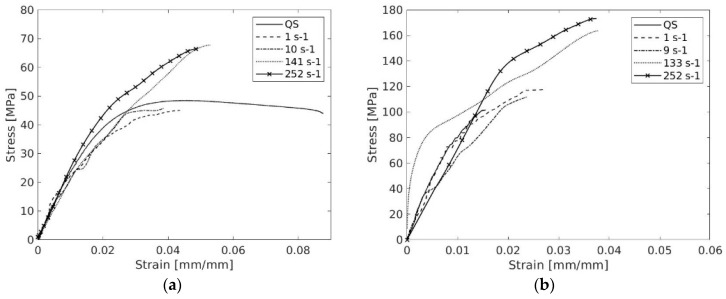
(**a**) Summary of strain−stress curves for different tested strain rate: (**a**) DCPD and (**b**) GRFP.

Figure 12 summarizes the results obtained for all the studied rates for both materials, and Table 24 summarizes the mechanical properties for the mechanical characterization tensile tests for DCPD and GRFP.

A noticeable strain rate dependency was observed for tensile strength and failure strain values for both materials. Focusing on DCPD, it is observed that increasing the strain rate up to 10 s^−1^ does not directly affect the maximum stress peak; however, fracture occurs with less elongation. When scaled to higher strain rates, the maximum stress values tend to increase considerably with higher elongation, while maintaining the negligible difference in maximum stress between 150 s^−1^ and 250 s^−1^.

Regarding GRFP, the strain rate has a significant impact on the strain–stress results. Similarly, stiffness remains similar for all cases, but some differences are observed in ultimate stress and failure strain. The strain rate sensitivity is positive and aligns with the literature. However, a direct comparison with documented values is not suitable due to variations in the manufacturing process. Furthermore, the failure strain tends to increase with the increasing strain rate. In general, for both materials, no significant differences in the failure modes were observed as the deformation rate increased, except that the behaviour became more pronounced.

The specimen’s toughness is calculated based on the area under the obtained stress–strain curves. Table 25 summarizes the obtained toughness for the conducted tests. In the QS tests, DCPD exhibits significantly higher toughness capacity than the GRFP. However, at higher strain rates, GRFP demonstrates a greater ability to absorb energy before failure. For strain rates on the order of 10 s^−1^, the obtained values are similar. In the SHPB tests, the GRFP toughness is approximately twice that observed in DCPD.

## 5. Conclusions

This study focused on determining the mechanical properties of two materials present in a coach section subjected to frontal collisions, DCPD panels in their neat state and glass-fibre-reinforced polymer (GRFP). Additionally, as a collision is a dynamic event, it is important to determine the effect of the strain rate on the materials. This characterization was based on tensile loads. The tests were conducted simultaneously using three different machines to achieve strain rates ranging from QS to 250 s^−1^. The dynamic results reached average strain rates of about 1 s^−1^ and 10 s^−1^ for both materials and achieved average strain rates of about 140 s^−1^ and 262 s^−1^ for DCPD and 128 s^−1^ and 252 s^−1^ for GRFP, respectively, employing SHPB.

DCPD properties are scarcely documented, making this study one of the first to explore its high strain rate behaviour. Reported QS values of 2087 MPa for tensile modulus and 47.73 MPa for ultimate strength align with those documented in the literature (tensile modulus of 1500–1870 MPa and tensile strength of 38–53 MPa). Another important aspect to mention is the overlap of elastic behaviour at any studied rate, indicating a similarity in stiffness. In the tensile tests for DCPD, the test fixture effectively characterized the material, revealing a valid failure mode characterized by multiple surface-transverse fissures within microseconds.

Both materials exhibit positive strain rate sensitivity in tensile strength and failure strain. The strain rate in DCPD has little impact on the peak stress but reduces elongation up to 10 s^−1^. At higher strain rates, both the maximum stress and elongation increase significantly, with minimal differences between 150 s^−1^ and 250 s^−1^. In GFRP, the strain rate considerably influences the stress–strain response, particularly in ultimate stress and failure strain, while stiffness remains relatively constant. Regarding failure modes, no significant differences were observed with increasing strain rate, except more pronounced behaviour at higher rates.

The aim was the evaluation of both materials with respect to their use in the automotive industry. According to the obtained toughness, it can be seen that for QS, DCDP has a greater capacity to absorb energy, which is positive in terms of crashworthiness. However, in such an event, the strain rates would be much higher, and in that case, the trend is reversed, with GFRP showing a better response in retaining energy. The results indicate that, although these materials are widely used in real-world automotive applications, they are not suitable for primary structural reinforcement. Although, when combined with other structural solutions, they can provide added value, with GFRP proving more advantageous than DCPD for higher strain rates.

## Figures and Tables

**Table 1:** Some of the mechanical properties of the different glass in glass fibre production.

	Glass Type	E	C	D	S	R
Property						
Density [g/cm^3^]		2.54	2.49	2.16	2.49	2.49
Young Modulus—20 °C [GPa]		73.50	70.0	52.50	86.50	86.50
Tensile Strength—20 °C [GPa]		3.50	2.80	2.45	3.50	4.65
Elongation [%]		4.50	4.00	4.50	5.30	5.30

**Table 2:** Some properties of different fibres with different polymer combinations.

Composite	Impact Strength [kJ/m^2^]	Impact Energy [J]
E-glass Fibre (70 wt%) + Epoxy [25]	151	-
Carbon Fibre + Epoxy [20]	38	-
Aramid Fibres + Epoxy [20]	134	-
2/3 Carbon Fibre + 1/3 Aramid Fibres + Epoxy [20]	87	-
1/3 Carbon Fibre + 2/3 Aramid Fibres + Epoxy [20]	110	-
E-glass Fibre 0–90° + Epoxy [26]	-	14.7
E-glass Fibre 0–90° + Epoxy [21]	-	11.4
E-glass Fibre 0–90° + Epoxy [18]	500	-
Carbon Fibre 0–90° + Epoxy [18]	950	-
E-glass Fibre + Polyester [22]	155	-
E-glass Fibre (20 wt%)) + Polyester [23]	-	41.7
E-glass Fibre 90° + Polyester [24]	-	37.9
E-glass Fibre 45° + Polyester [24]	-	41.4
E-glass Fibre 0° + Polyester [24]	-	41.7

**Table 3:** Typical RIM process parameters.

Temperature in the mould	30–70 [°C]
Temperature of the chemical substances	30–40 [°C]
Clamping force, e.g., automotive bumpers	100–150 [tonne]
Mixing head pressure	10.3–20.6 [MPa]
In-mould pressure	0.4–1.1 [MPa]
Viscosity of the material	0.1 × 10^−6^–1 × 10^−6^ [MPa.s]
Demoulding time	30–60 [s]

**Table 4:** Technical data from Telene^®^ for different series.

	Series	1620	1650	1651
Property				
Specific gravity [g/cc]		1.03	1.03	1.03
Young’s modulus [GPa]		1.87	1.87	−
Tensile strength [MPa]@yield		43	43	44
Elongation [%]@yield		5	5	−
Flexural strength [MPa]		67	67	67
Flexural modulus [MPa]		1850	1850	1850
Poisson’s ratio		−	0.39	−

**Table 5:** Properties of the cured resin [55].

Property	Value	Unit
Tensile strength	41	[MPa]
Elongation	2.1	[%]
Flexural strength	105	[MPa]
Barcol hardness	35	[BHN]
Contraction in the cure	8.6	[%]

**Table 6:** Properties of the glass fibre [56].

Property	Value	Unit
Weight	450 ± 8%	[gr/m^2^]
Tensile breaking force (machine direction—MD)	≥120	[N]
Tensile breaking force (cross − machine direction—CD)	≥100	[N]

**Table 7:** GFRP specimen geometry of each material’s typology according to ASTM D3039 [60].

Specimen Geometry	Length [mm]	Width [mm]	Thickness [mm]	Tabs
Standard	250	25	2.5	Yes/No
Non-Standard	200	25	3.5	Yes

**Table 8:** DCPD specimen geometry of each material’s typology according to ASTM D638 [61].

Specimen Geometry	Overall Length [mm]	Length—Narrow Section [mm]	Overall Width [mm]	Width—Narrow section [mm]	Radius of the Fillet [mm]	Grips Distance [mm]
Standard	165	57	19	13	76	115
Non-Standard	185	57	19	13	76	115

**Table 9:** Setup parameters for optic measurements for QS loading condition.

Lens (Extension)	Virtual Extensometer [mm]	Acquisition Interval [fps]	Work Distance [m]	Aperture Setting	Pixel Size [px/mm]	Subset Size [px]
Rodagon 60 mm	20	20	0.4	f/5.6	21	7

**Table 10:** Setup parameters for optic measurements for dynamic loading condition—HSM.

Lens (Extension)	Exposure Time [ms]	Virtual Extensometer [mm]	Acquisition Interval [fps]	Work Distance [m]	Aperture Setting	Pixel Size [px/mm]	Subset Size [px]
Nikon 60 mm Micro AF-D	0.05	8	6400	0.35	f/2.8	16	7

**Table 11:** Setup parameters for optic measurements for dynamic loading condition—Hopkinson bar.

Lens (Extension)	Image Resolution [px^2^]	Virtual Extensometer [mm]	Acquisition Interval [fps]	Work Distance [m]	Aperture Setting	Pixel Size [px/mm]	Subset Size [px]	Shutter Speed [sec]
Nikon 60 mm Micro AF-D	256 × 128	8	120,000	0.35	f/2.8	16	7	1/60,000

**Table 12:** Tensile behaviour of the DCPD polymer under the quasi-static loading.

	Specimen	001	002	003	004	005	006	$\bar{X}$	CV
Property									
Elastic Modulus [MPa]		2036	2087	1945	2245	1953	2019	2053.2	5.96%
Ultimate Strength [MPa]		48.50	47.73	48.49	49.11	48.95	46.64	48.56	1.11%
Failure Strain [%]		8.40%	8.76%	11.40%	6.70%	8.82%	3.00%	8.82%	19.09%

**Table 13:** Mechanical properties of the GFRP for QS tensile testing.

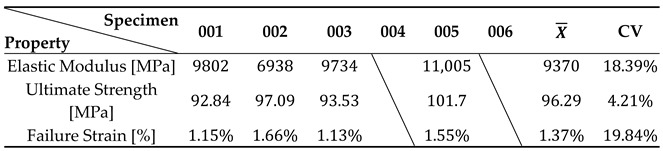

**Table 14:** Dimensions of the new dog-bone geometry specimen, for GRFP material, expressed in millimetres.

Overall Length [mm]	Length—Narrow Section [mm]	Overall Width [mm]	Width—Narrow Section [mm]	Radius of the Fillet [mm]
200	60	25	17	60

**Table 15:** Mechanical properties of the glass fibre/polyester polymer tensile test dog-bone geometry.

	Specimen	001	002	003	004	005	$\bar{X}$	CV
Property								
Elastic Modulus [MPa]		6710	6820	7251	8101	6313	6774	5.69%
Ultimate Strength [MPa]		109.30	110.90	108.14	118.90	97.90	106.56	5.52%
Failure Strain [%]		1.77%	1.98%	1.60%	1.63%	1.60%	1.74%	10.40%

**Table 16:** Tensile behaviour of the DCPD polymer for 1st set of tests under intermediate strain rate loading.

	Specimen	001	002	003	004	005	$\bar{X}$	CV
Property								
Elastic Modulus [MPa]		2198	1959	1818	2110	1843	1985	8.34%
Ultimate Strength [MPa]		45.73	47.85	42.81	45.49	45.05	45.38	3.97%
Failure Strain [%]		3.41%	4.37%	4.46%	3.86%	4.36%	4.09%	10.95%

**Table 17:** Tensile behaviour of the DCPD polymer for 2nd set of tests under intermediate strain rate loading.

	Specimen	001	002	003	004	005	$\bar{X}$	CV
Property								
Elastic Modulus [MPa]		1882	2123	1832	1903	2143	1977	7.35%
Ultimate Strength [MPa]		46.87	50.29	47.27	48.32	45.71	47.69	3.63%
Failure Strain [%]		3.72%	5.58%	3.53%	3.68%	3.86%	4.07%	20.94%

**Table 18:** Tensile behaviour of the GRFP for 1st set of tests under intermediate strain rate.

	Specimen	001	002	003	004	005	$\bar{X}$	CV
Property								
Elastic Modulus [MPa]		10,634	9482	8251	10,975	11,451	10,158	12.70%
Ultimate Strength [MPa]		117.04	117.61	109.52	117.89	101.45	112.70	6.38%
Failure Strain [%]		2.29%	2.70%	2.96%	2.93%	1.93%	2.56%	17.31%

**Table 19:** Tensile behaviour of the GRFP for 2nd set of tests under intermediate strain rate loading.

	Specimen	001	002	003	004	005	$\bar{X}$	CV
Property								
Elastic Modulus [MPa]		9957	9296	11,820	10,594	10,234	10,380	9.00%
Ultimate Strength [MPa]		111.63	105.48	104.74	134.72	129.13	117.14	11.87%
Failure Strain [%]		2.36%	2.64%	2.52%	2.95%	2.01%	2.50%	13.91%

**Table 20:** Tensile behaviour of the DCPD polymer under high strain rate loading (1st set of tests).

	Specimen	001	002	003	004	005	$\bar{X}$	CV
Property								
Elastic Modulus [MPa]		2109	2237	2775	3371	1918	2482	23.78%
Ultimate Strength [MPa]		67.73	65.66	66.20	63.24	63.66	65.30	2.84%
Failure Strain [%]		5.29%	4.68%	4.78%	4.54%	5.48%	4.95%	8.24%

**Table 21:** Tensile behaviour of the DCPD polymer under high strain rate loading (2nd set of tests).

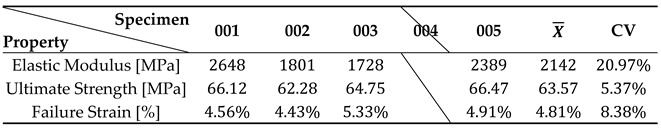

**Table 22:** Tensile behaviour of the GRFP under high strain rate loading (1st set of tests).

	Specimen	001	002	003	004	005	$\bar{X}$	CV
Property								
Elastic Modulus [MPa]		4326	4532	3952	4084	4600	4299	6.50%
Ultimate Strength [MPa]		163.53	164.52	173.91	167.84	158.70	165.70	3.40%
Failure Strain [%]		3.78%	3.63%	4.40%	4.11%	3.45%	3.87%	9.83%

**Table 23:** Tensile behaviour of the GRFP under intermediate strain rate loading (2nd set of tests).

	Specimen	001	002	003	004	005	$\bar{X}$	CV
Property								
Elastic Modulus [MPa]		6267	5721	5485	7037	7288	6359	12.43%
Ultimate Strength [MPa]		150.46	147.28	172.89	157.05	173.29	160.19	7.67%
Failure Strain [%]		3.45%	3.72%	4.32%	3.51%	3.75%	3.75%	9.17%

**Table 24:** Summary of mechanical properties for mechanical characterization tensile tests for DCPD and GRFP.

	DCPD					GRFP				
	QS	1 s^−1^	10 s^−1^	141 s^−1^	252 s^−1^	QS	1 s^−1^	9 s^−1^	133 s^−1^	252 s^−1^
Elastic Modulus [MPa]	2087	1843	2143	2109	2389	11,005	9482	9957	4532	7288
Ultimate Strength [MPa]	47.73	45.05	45.71	67.73	66.47	101.7	117.61	111.63	164.52	173.29
Failure Strain [%]	8.76	4.36	3.86	5.29	4.91	1.55	2.70	2.36	3.63	3.75

**Table 25:** Summary of toughness of both materials according to the tensile tests for DCPD and GRFP to the tested strain rates, in [MJ/m^3^].

DCPD					GFRP				
QS	1 s^−1^	10 s^−1^	141 s^−1^	252 s^−1^	QS	1 s^−1^	9 s^−1^	133 s^−1^	252 s^−1^
3.592	1.390	1.190	2.158	2.130	0.980	2.212	1.630	4.453	4.202

## Data Availability

The relevant data generated and analysed in the current study are available from the corresponding author upon reasonable request.

## References

[1] Lopes R., Ramos N.V., Cunha R., Maia R., Rodrigues R., Parente M.P.L., Moreira P.M.G.P. (2023). Coach crashworthiness and failure analysis during a frontal impact. Eng. Fail. Anal..

[2] Lopes R., Ramos N.V., Cunha R., Maia R., Rodrigues R., Parente M.P.L., Moreira P.M.G.P. (2022). Passive Safety Solutions on Coach according ECE R29: Experimental and Numerical analyses. Procedia Struct. Integr..

[3] Teixeira A., Ribeiro B. (2010). Use of DCPD-RIM on exterior panels for the automotive industry. Rapid Prod. Dev..

[4] European Commission (2019). ECE-R29. Regulation No 29 of the Economic Commission for Europe of the United Nations (UN/ECE)—Uniform provisions concerning the approval of vehicles with regard to the protection of the occupants of the cab of a commercial vehicle [2019/1850]. Off. J. Eur. Union.

[5] Lopes R., Pinto S.M., Parente M.P.L., Moreira P.M.G.P., Baptista A.J. (2024). Crashworthiness optimisation and environmental impact assessment of a redesigned passenger coach integrating lean design-for-X framework. J. Eng. Des..

[6] Silva C.J.G., Lopes R.F.F., Domingues T.M.R.M., Parente M.P.L., Moreira P.M.G.P. (2024). Crashworthiness topology optimisation of a crash box to improve passive safety during a frontal impact. Struct. Multidiscip. Optim..

[7] Duan S., Tao Y., Han X., Yang X., Hou S., Hu Z. (2014). Investigation on structure optimization of crashworthiness of fiber reinforced polymers materials. Compos. Part B Eng..

[8] Li Z., Yu Q., Zhao X., Yu M., Shi P., Yan C. (2017). Crashworthiness and lightweight optimization to applied multiple materials and foam-filled front end structure of auto-body. Adv. Mech. Eng..

[9] Wan M. (2011). Different Types of Chassis: Glass-Fiber Body. AutoZine Technical School. http://www.autozine.org/technical_school/chassis/tech_chassis2.htm#Glass-Fiber.

[10] Blanco S. (2010). Fisker Karma’s Aluminum Space Frame Digitally Revealed. https://www.autoblog.com/2010/03/01/fisker-karmas-aluminum-space-frame-digitally-revealed/.

[11] Chen Y., Cheng X., Fu K. (2020). Multi-material design of a vehicle body considering crashworthiness safety and social effects. Int. J. Crashworthiness.

[12] Pravilonis T., Sokolovskij E. (2021). Steel and Hybrid Bus Safety Frame: The Analysis of Deformation. Reliability and Statistics in Transportation and Communication.

[13] Stauber R., Vollrath L. (2007). Plastics in Automotive Engineering: Exterior Applications.

[14] Masuelli M.A., Masuelli M.A. (2013). Introduction of Fibre-Reinforced Polymers—Polymers and Composites: Concepts, Properties and Processes. Fiber Reinforced Polymers.

[15] Yang L., Liao Z., Qiu C., Hong Z., Yang J. (2024). Experimental study on the impact resistance and damage tolerance of thermoplastic FMLs. Thin-Walled Struct..

[16] Gupta M. (2007). Polymer Composite.

[17] Kausar A. (2017). Role of thermosetting polymer in structural composite. Am. J. Polym. Sci. Eng..

[18] Ratna D. (2008). Toughened FRP composites reinforced with glass and carbon fiber. Compos. Part A Appl. Sci. Manuf..

[19] Zhang M., Matinlinna J.P. (2012). E-Glass Fiber Reinforced Composites in Dental Applications. Silicon.

[20] Dorey G., Sidey G.R., Hutchings J. (1978). Impact properties of carbon fibre/Kevlar 49 fibre hydrid composites. Composites.

[21] Sanjay M.R., Yogesha B. (2016). Studies on mechanical properties of jute/E-glass fiber reinforced epoxy hybrid composites. J. Miner. Mater. Charact. Eng..

[22] Elahi A., Hossain M.M., Afrin S., Khan M.A. Study on the mechanical properties of glass fiber reinforced polyester composites. Proceedings of the International Conference on Mechanical Industrial and Energy Engineering.

[23] Gupta G., Gupta A., Dhanola A., Raturi A. (2016). Mechanical behavior of glass fiber polyester hybrid composite filled with natural fillers. IOP Conf. Ser. Mater. Sci. Eng..

[24] Alam S., Habib F., Irfan M., Iqbal W., Khalid K. (2010). Effect of orientation of glass fiber on mechanical properties of GRP composites. J. Chem. Soc. Pak..

[25] Mohan Kumar S., Raghavendra Ravikiran K., Govindaraju H.K. (2018). Development of E-Glass Woven Fabric/Polyester Resin Polymer Matrix Composite and Study of Mechanical Properties. Mater. Today Proc..

[26] Ho M.-P., Lau K.-T. (2012). Design of an impact resistant glass fibre/epoxy composites using short silk fibres. Mater. Des..

[27] Daliri V., Zeinedini A. (2019). Flexural Behavoiur of the Composite Sandwich Panels with Novel and Regular Corrugated Cores. Appl. Compos. Mater..

[28] Eriksen R.N.W. (2014). High Strain Rate Characterisation of Composite Materials.

[29] Körber H. (2010). Mechanical Response of Advanced Composites Under High Strain Rates. Ph.D. Thesis.

[30] Chen W., Meng Q., Hao H., Cui J., Shi Y. (2017). Quasi-static and dynamic tensile properties of fiberglass/epoxy laminate sheet. Constr. Build. Mater..

[31] Zhang X., Wang H., Lv Y., Li J. (2022). Quasi-static and dynamic properties of unidirectional glass fiber/epoxy laminate for structural strengthening. Mater. Struct..

[32] Naik N.K., Yernamma P., Thoram N.M., Gadipatri R., Kavala V.R. (2010). High strain rate tensile behavior of woven fabric E-glass/epoxy composite. Polym. Test..

[33] Shokrieh M.M., Omidi M.J. (2009). Tension behavior of unidirectional glass/epoxy composites under different strain rates. Compos. Struct..

[34] Barré S., Chotard T., Benzeggagh M.L. (1996). Comparative study of strain rate effects on mechanical properties of glass fibre-reinforced thermoset matrix composite. Compos. Part A Appl. Sci. Manuf..

[35] Ou Y., Zhu D. (2015). Tensile behavior of glass fiber reinforced composite at different strain rates and temperatures. Constr. Build. Mater..

[36] Ahmed A., Zillur Rahman M., Ou Y., Liu S., Mobasher B., Guo S., Zhu D. (2021). A review on the tensile behavior of fiber-reinforced polymer composites under varying strain rates and temperatures. Constr. Build. Mater..

[37] Telene SAS Values Data Sheet Telene.

[38] Camboa A.M.S. (2016). Development of Lightweight and Cost-Efficient Exterior Body Panels for Electric Vehicles. Ph.D. Thesis.

[39] Zhang Z., Yang Z., Zhou F., He X. (2022). Effect of different catalyst ratios on the ring-opening metathesis polymerization (ROMP) of dicyclopentadiene. Polyolefins J..

[40] David A., Huang J., Richaud E., Yves Le Gac P. (2020). Impact of thermal oxidation on mechanical behavior of polydicylopentadiene: Case of non-diffusion limited oxidation. Polym. Degrad. Stab..

[41] Yoo H.M., Kwon D.-J., Park J.-M., Yum S.H., Lee W.I. (2017). Mechanical properties of norbornene-based silane treated glass fiber reinforced polydicyclopentadiene composites manufactured by the S-RIM process. E-Polymers.

[42] Elkington M., Bloom D., Ward C., Chatzimichali A., Potter K. (2015). Hand layup: Understanding the manual process. Adv. Manuf. Polym. Compos. Sci..

[43] Camboa A., Ribeiro B.D., Nunes J.P., Alves J.L. (2013). A Mechanical Analysis of Polydicyclopentadiene with Metal Inserts Through Flexural Load.

[44] Yang Y.-S., Lafontaine E., Mortaigne B. (1997). Curing study of dicyclopentadiene resin and effect of elastomer on its polymer network. Polymer.

[45] Rosato D.V., Rosato D.V., Rosato M.V. (2004). Plastic Product Material and Process Selection Handbook.

[46] Snyder C.D. Materials for Reaction Injection Molding (RIM) Processing. Proceedings of the 2001 Annual Conference of the Composites Fabricators Association.

[47] Ng H. (1992). Studies of Reactive Polymer Processing for Dicyclopentadiene RIM and Filled Epoxy Systems. Ph.D. Thesis.

[48] Oliveira J. (2014). Estudo e Otimização do Processo RIM Para DCPD. Master’s Thesis.

[49] Palardy G., Hubert P., Haider M., Lessard L. (2008). Optimization of RTM processing parameters for Class A surface finish. Compos. Part B Eng..

[50] Camboa A., Silva H., Teixeira A., Ribeiro B., Nunes J.P. (2012). Hybrid Design for Automotive Body Panels.

[51] Bocian M., Pach J., Jamroziak K., Kosobudzki M., Polak S., Pyka D., Kurzawa A., Kurowski J. (2017). Experimental and numerical analysis of aramid fiber laminates with DCPD resin matrix subjected to impact tests. MATEC Web Conf..

[52] Vallons K.A.M., Drozdzak R., Charret M., Lomov S. Exploratory study on the behaviour of glass/pdcpd composites. Proceedings of the 20th International Conference on Composite Materials.

[53] Telene SAS (2007). Telene the Cost Effective Solution for Large Parts from Low to High Volumes.

[54] (2023). Textile-Glass-Reinforced Plastics—Prepregs, Moulding Compounds and Laminates—Determination of the Textile-Glass and Mineral-Filler Content Using Calcination Methods.

[55] Ecocompositos-Ecoresins (2013). Brochure ECORESIN 0166 HDT.

[56] Ecocompositos (2024). Brochure Ecomat 450 CNW (Emulsion) 450 gr/m^2^ 1250 mm.

[57] Telene® (2017). Safety Data Sheet TELENE © 16XX/26XX A.

[58] Telene SAS (2018). Telene Process Book.

[59] Lopes R.F.F. (2024). Development of New Structural Solutions for the Automotive Industry.

[60] (2000). Standard Test Method for Tensile Properties of Polymer Matrix Composite Materials.

[61] (2010). Standard Test Method for Tensile Properties of Plastics.

[62] Correlated Solutions (2009). VIC-2D, Testing Guide.

[63] Sousa P.J., Eslami S., Direito F.P., Moreira P.M.G.P. (2022). Development of a small-scale testing machine for use with interferometric monitoring methods. Procedia Struct. Integr..

[64] Rockwell Automation MPL-B680H-MJ74AA. Allen-Bradley. https://www.rockwellautomation.com/en-nz/products/details.MPL-B680H-MJ74AA.html.

[65] Ab V.W. Dynamometer TC4—Stainless Steel, 50kN—Vetek. https://www.vetek.com/en/article/dynamometer-tc4-stainless-steel-50kn.

[66] Gsvitec (1981). Multiled MX—Technical Data Sheet.

[67] Smerd R., Winkler S., Salisbury C., Worswick M., Lloyd D., Finn M. (2005). High strain rate tensile testing of automotive aluminum alloy sheet. Int. J. Impact Eng..

[68] Shamchi S.P. (2021). High Strain Rate Behavior of Aerospace Materials. Ph.D. Thesis.

[69] Ledford N., Paul H., Ganzenmüller G., May M., Höfemann M., Otto M., Petrinic N. (2015). Investigations on specimen design and mounting for Split Hopkinson Tension Bar (SHTB) experiments. EPJ Web Conf..

[70] Sharma S., Chavan V.M., Agrawal R.G., Patel R.J., Kapoor R., Chakravartty J.K. (2011). Split-Hopkinson Pressure Bar: An Experimental Technique for High Strain Rate Tests.

[71] Gama B.A., Lopatnikov S.L., Gillespie J.W. (2004). Hopkinson bar experimental technique: A critical review. Appl. Mech. Rev..

[72] Constantino S.C. (2016). Aluminium FSW Joints Under High Strain Rate Tensile Testing. Master’s Thesis.

[73] Shamchi S.P., Queirós de Melo F.J.M., Tavares P.J., Moreira P.M.G.P. (2019). Thermomechanical characterization of Alclad AA2024-T3 aluminum alloy using split Hopkinson tension bar. Mech. Mater..

[74] Shamchi S.P., Singh A., Sguazzo C., Zhao Z., Yi X., Tavares P.J., Moreira P.M.G.P. (2020). Strain-rate sensitivity of electrically modified carbon/epoxy composites under dynamic compressive loading. Procedia Struct. Integr..

[75] Sousa P.J., Lopes R., Reis J.M., Moreira P.M.G.P. (2022). Custom control system for Split Hopkinson Pressure bars. Procedia Struct. Integr..

[76] Shamchi S., Yi X., Moreira P.M.G.P. (2023). Strain-Rate Dependence of Electrically Modified Unidirectional Carbon/epoxy Laminates Under In-plane Tensile Loading. Appl. Compos. Mater..

[77] Mascarenhas W.N., Ahrens C.H., Ogliari A. (2004). Design criteria and safety factors for plastic components design. Mater. Des..

[78] Chougrani K., Gisolf A., Dijkstra F. Detection of transparent cracks using nonlinear acoustics. Proceedings of the 18th World Conference on NDT.

[79] Fazlali B., Lomov S.V., Swolfs Y. (2024). Reducing stress concentrations in static and fatigue tensile tests on unidirectional composite materials: A review. Compos. Part B Eng..

[80] El-Wazery M.S., El-Elamy M.I., Zoalfakar S.H. (2017). Mechanical Properties of Glass Fiber Reinforced Polyester Composites. Int. J. Appl. Sci. Eng..

[81] Schoßig M., Bierögel C., Grellmann W., Mecklenburg T. (2008). Mechanical behavior of glass-fiber reinforced thermoplastic materials under high strain rates. Polym. Test..

[82] Ou Y., Zhu D., Zhang H., Huang L., Yao Y., Li G., Mobasher B. (2016). Mechanical Characterization of the Tensile Properties of Glass Fiber and Its Reinforced Polymer (GFRP) Composite under Varying Strain Rates and Temperatures. Polymers.

[83] Milisavljević J., Petrović E., Ćirić I., Mančić M., Marković D., Đorđević M. (2012). Tensile testing for different types of polymers. Danubia-Adria Symposium.

[84] Neville A.M., Dilger W.H., Brooks J.J. (1983). Creep of Plain and Structural Concrete.

